# P-1151. The in vitro activity of ceftazidime-avibactam against Enterobacterales isolates collected in Latin America as a part of the ATLAS Global Surveillance Program 2019-2023, by β-lactamase carriage

**DOI:** 10.1093/ofid/ofaf695.1345

**Published:** 2026-01-11

**Authors:** Mark Estabrook, Henry Li, Gregory Stone, Katherine Perez, Paurus Irani, Daniel F Sahm

**Affiliations:** IHMA, Schaumburg, IL; IHMA, Schaumburg, IL; Pfizer, Inc., Groton, Connecticut; Pfizer, Inc., Groton, Connecticut; Pfizer United Kingdom, London, England, United Kingdom; IHMA, Schaumburg, IL

## Abstract

**Background:**

Ceftazidime is a third-generation cephalosporin that is available in combination with the diazabicyclooctane β-lactamase inhibitor avibactam for the treatment of complicated intra-abdominal infections, complicated urinary tract infections, and hospital/ventilator-acquired bacterial pneumonia caused by susceptible Gram-negative pathogens. Here we show the *in vitro* activity of ceftazidime-avibactam (CAZ-AVI) against Enterobacterales collected in Latin America for the ATLAS surveillance program (2019-2023), stratified by β-lactamase carriage.

**Methods:**

14,792 isolates from 37 medical centers in 10 countries were collected and tested for susceptibility using the broth microdilution method according to CLSI guidelines. Analysis was performed with CLSI 2025 breakpoints. Isolates testing with meropenem MIC values >1 µg/mL or a subset of *Escherichia coli, Klebsiella pneumoniae, K. oxytoca,* or *Proteus mirabilis* isolates testing with ceftazidime and/or aztreonam MIC values >2 µg/mL were screened for β-lactamase genes by PCR, which were sequenced when identified.

**Results:**

Isolates that carried metallo-β-lactamases (MBLs) were not susceptible to CAZ-AVI or meropenem (MEM) and were comprised mostly of NDM- (95%) or VIM-producers (3%) (Table 1 and Figure 1). Of isolates with serine-carbapenemases, 99.3% were susceptible to CAZ-AVI, while only 6.4% were susceptible to MEM. KPC was the sole carbapenemase identified in 94% of these isolates, with OXA-48 in 5%. Of 91 isolates carrying AmpC and no carbapenemase, 97.8% were susceptible to CAZ-AVI and 96.7% were susceptible to MEM. These isolates primarily carried variants of CMY (74%) or DHA (20%). Of isolates that carried an ESBL and no carbapenemase or AmpC, 99.7% were susceptible to CAZ-AVI and 93.3% were susceptible to MEM. The majority of these isolates carried a variant of CTX-M and no other family of ESBL (96%).

**Conclusion:**

Ceftazidime-avibactam retained potent *in vitro* activity against isolates that carry β-lactamases other than MBLs in this collection of molecularly characterized Enterobacterales isolates collected in Latin America from 2019-2023.

**Disclosures:**

Katherine Perez, PhD, Pfizer: Stocks/Bonds (Public Company) Paurus Irani, MD, Pfizer, Inc.: Employee|Pfizer, Inc.: Stocks/Bonds (Private Company)

